# *TaMIP1* gene breaks the dilemma of coordinating drought resistance and high yield in wheat

**DOI:** 10.1093/plphys/kiag451

**Published:** 2026-06-27

**Authors:** Zihan Xu, Rongxuan Zhang, Ning Zhang

**Affiliations:** State Key Laboratory of Wheat Improvement, College of Agronomy, Shandong Agricultural University, Tai’an, Shandong 271018, China; State Key Laboratory of Wheat Improvement, College of Agronomy, Shandong Agricultural University, Tai’an, Shandong 271018, China; State Key Laboratory of Wheat Improvement, College of Agronomy, Shandong Agricultural University, Tai’an, Shandong 271018, China; Assistant Features Editor, Plant Physiology, American Society of Plant Biologists

As climate change intensifies the frequency and severity of drought episodes, improving crop resilience while sustaining productivity has become one of the central challenges facing global agriculture (Lobell et al. 2011). Wheat, which provides approximately 25% of the calories consumed worldwide, is particularly vulnerable to water limitation during critical developmental stages. Roots are the first organs to encounter soil water deficits and play a decisive role in determining plant performance under drought (Fang et al. 2019). Consequently, understanding the genetic mechanisms that shape root system architecture has emerged as a major focus of crop improvement efforts. Yet breeding for larger or denser root systems is complicated by a longstanding biological dilemma: investments in root growth can enhance water acquisition and stress tolerance but often come at the expense of resources available for grain production (Sciara et al. 2025).

Recently, Fan et al. (2026) identified a drought-responsive transcription factor, *TaMIP1*, that links root density, drought adaptation, and grain yield in wheat. By integrating drought-induced transcriptomic profiling with natural variation analysis, the authors discovered 2 *TaMIP1* haplotypes associated with root dry weight and developed a derived Cleaved Amplified Polymorphic Sequence (dCAPS) molecular marker for their identification. Functional characterization revealed that *TaMIP1* encodes a nuclear-localized bHLH transcription factor. CRISPR-Cas9-generated *Tamip1* mutants exhibited reduced root number and root biomass, whereas overexpression lines of *TaMIP1* in rice increased root development, demonstrating a conserved role in regulating root density across monocots. Mechanistically, TaMIP1 interacts with the root-development regulator TaMOR, an LBD transcription factor, and functions as a transcriptional activator that directly binds E-box cis-elements in the promoters of genes involved in auxin signaling, ABA responses, root development, and drought adaptation (Fig. 1). Transcriptomic analyses further established *TaMIP1* as a central regulatory node coordinating developmental and stress-response pathways. Notably, co-expression of *TaMIP1* and *TaMOR* produced additive activation of downstream targets, highlighting a cooperative regulatory module controlling root system architecture. Beyond elucidating this molecular mechanism, the study uncovered a physiological trade-off mediated by *TaMIP1*: reduced root density enhanced grain yield under well-watered conditions but compromised drought resistance, whereas increased root density improved drought adaptation at a potential cost to yield. Importantly, the superior haplotype, *Hap-*2A-2, displayed stronger transactivation activity and conferred greater root biomass and drought tolerance. By combining favorable alleles of *TaMIP1* and *TaMOR*, the authors further identified an elite haplotype combination that simultaneously promotes robust root growth and desirable spike traits, providing a promising strategy to mitigate the long-recognized trade-off between productivity and stress resilience.

**Figure 1:**
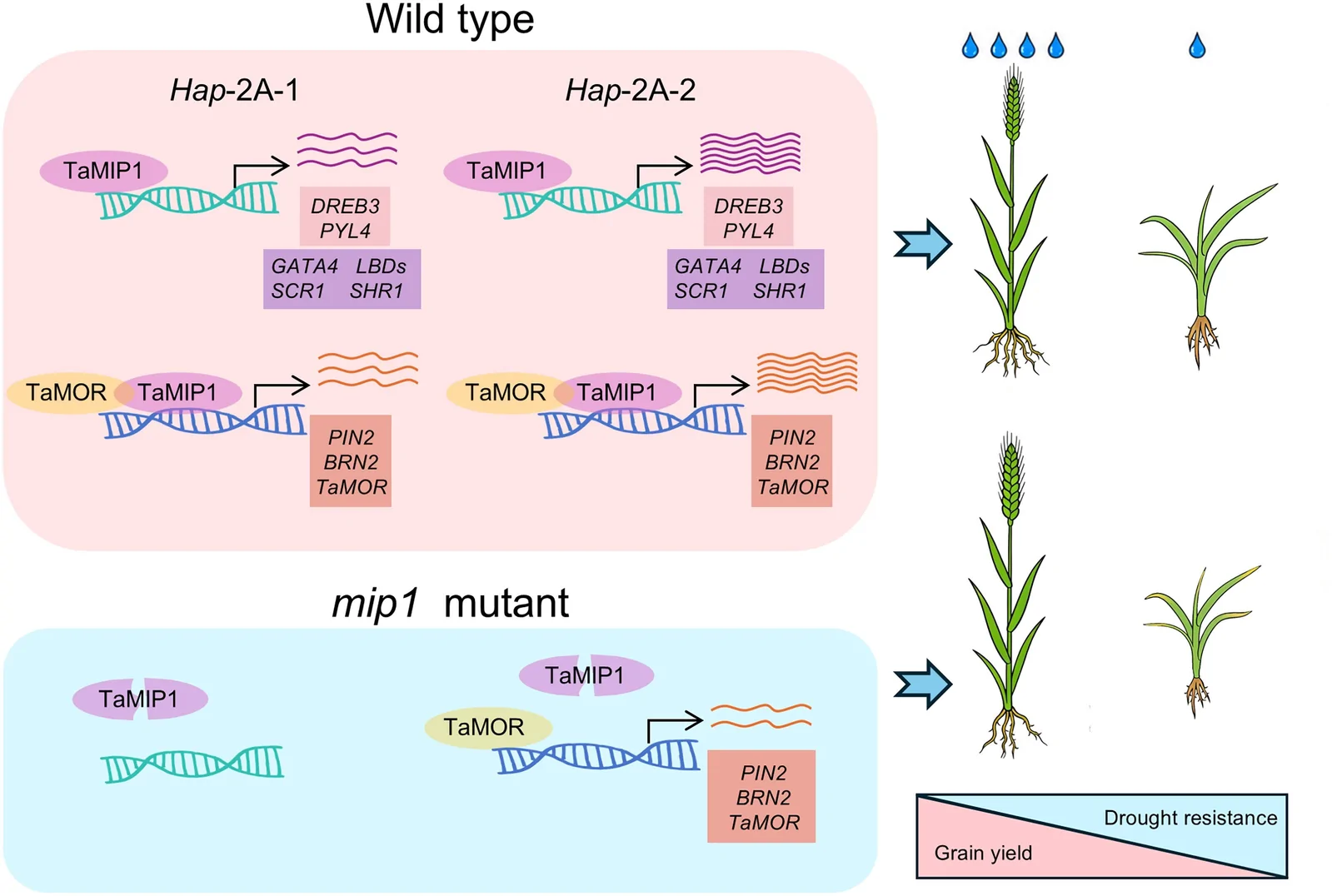
Proposed model for TaMIP1-mediated regulation of root density and the trade-off between drought resistance and grain yield in wheat (summarized from Fan et al. 2026). The superior haplotype *Hap-*2A-2 exhibits stronger transactivation activity than *Hap-*2A-1, enhancing the expression of drought-responsive and root-development genes. TaMIP1 interacts with TaMOR to cooperatively activate downstream targets involved in root formation, thereby increasing root density and drought tolerance. In contrast, loss of TaMIP1 function reduces root development and drought resistance but favors grain yield under well-watered conditions.

Beyond identifying a new regulator of root development, this study provides a conceptual advance in understanding how wheat balances drought adaptation and grain productivity. Previous studies uncovered genes controlling root architecture, but few connected root developmental pathways to the physiological trade-off between yield and stress tolerance (Li et al. 2022 and Ma et al. 2014). By identifying the *TaMIP1–TaMOR* regulatory module, Fan et al. (2026) establish a molecular framework linking auxin and ABA signaling, root density, and drought responses. Particularly noteworthy is the discovery that natural variation in *TaMIP1* affects both root biomass and agronomic performance, demonstrating how allelic diversity can shape adaptive strategies under contrasting water conditions. At the same time, the study opens several exciting avenues for future research. Root density represents only one dimension of root system architecture, and it will be important to determine how *TaMIP1* interacts with other root traits, such as rooting depth and water uptake efficiency, that contribute to drought adaptation. In addition, the molecular mechanisms by which drought stress suppresses *TaMIP1* expression while increased *TaMIP1* activity enhances drought tolerance remain unclear and warrant further investigation. Future studies should also evaluate the performance of elite *TaMIP1–TaMOR* haplotype combinations across diverse genetic backgrounds and environments. Such efforts will help translate the mechanistic insights gained from this work into breeding strategies capable of sustaining wheat productivity under increasingly variable climate conditions.

## Recent research articles in *Plant Physiology*

- Li et al. (2026) found that the R2R3-MYB transcription factor TaMYB96-2D promotes cuticular wax accumulation by directly activating diketone biosynthetic genes, thereby enhancing glaucousness and drought tolerance in wheat (https://doi.org/10.1093/plphys/kiaf668).
- Baca Cabrera et al. (2025) showed that more than a century of wheat breeding has progressively reduced root system size and hydraulic conductance, suggesting that modern cultivars have been inadvertently selected for more conservative water uptake strategies under high-input agricultural conditions (https://doi.org/10.1093/plphys/kiaf166).

## Data Availability

Data availability statement is not applicable.
